# Impact of the Italian Healthcare Outcomes Program (PNE) on the Care Quality of the Poorest Performing Hospitals

**DOI:** 10.3390/healthcare12040431

**Published:** 2024-02-07

**Authors:** Matteo Fiore, Alessandro Bianconi, Cecilia Acuti Martellucci, Annalisa Rosso, Enrico Zauli, Maria Elena Flacco, Lamberto Manzoli

**Affiliations:** 1 Department of Medical and Surgical Sciences, University of Bologna, 40126 Bologna, Italy; matteo.fiore7@studio.unibo.it (M.F.); alessandro.bianconi4@studio.unibo.it (A.B.); ceciliamartellucci@gmail.com (C.A.M.); 2 Department of Environmental and Prevention Sciences, University of Ferrara, 44121 Ferrara, Italy; annalisa.rosso@unife.it (A.R.); mariaelena.flacco@unife.it (M.E.F.); 3 Department of Medical Translation, University of Ferrara, 44121 Ferrara, Italy; enrico.zauli@unife.it

**Keywords:** healthcare quality, hospital care, quality assessment program, quality indicators, Italy

## Abstract

One of the main aims of the Italian National Healthcare Outcomes Program (Programma Nazionale Esiti, PNE) is the identification of the hospitals with the lowest performance, leading them to improve their quality. In order to evaluate PNE impact for a subset of outcome indicators, we evaluated whether the performance of the hospitals with the lowest scores in 2016 had significantly improved after five years. The eight indicators measured the risk-adjusted likelihood of the death of each patient (adjusted relative risk—RR) 30 days after the admission for acute myocardial infarction, congestive heart failure, stroke, chronic obstructive pulmonary disease, chronic kidney disease, femur fracture or lung and colon cancer. In 2016, the PNE identified 288 hospitals with a very low performance in at least one of the selected indicators. Overall, 51.0% (n = 147) of these hospitals showed some degree of improvement in 2021, and 27.4% of them improved so much that the death risk of their patients fell below the national mean value. In 34.7% of the hospitals, however, the patients still carried a mean risk of death >30% higher than the average Italian patient with the same disease. Only 38.5% of the hospitals in Southern Italy improved the scores of the selected indicators, versus 68.0% in Northern and Central Italy. Multivariate analyses, adjusting for the baseline performance in 2016, confirmed univariate results and showed a significantly lower likelihood of improvement with increasing hospital volume. Despite the overall methodological validity of the PNE system, current Italian policies and actions aimed at translating hospital quality scores into effective organizational changes need to be reinforced with a special focus on larger southern regions.

## 1. Introduction

Various forms of healthcare quality assessment systems have been used for decades to identify services with suboptimal performances and inefficiencies—priority targets for interventions are aimed at improving quality—and to stimulate the alignment of healthcare providers to the best standards of care [1,2].

The Centers for Medicare and Medicaid (CMS) in the USA, in particular, developed several monitoring, reporting and reward or penalty programs to improve the quality of healthcare services [3]. Penalties are also employed by the National Health Service of the United Kingdom [4], and by the Australian Institute of Health and Welfare [5]. Similarly, several other countries in the Organization for Economic Cooperation and Development (OECD) have implemented healthcare system evaluation models [6]. In Italy, the National Agency for Regional Healthcare Services (Agenzia Nazionale per i Servizi Sanitari Regionali, Agenas) established the Italian National Healthcare Outcomes Program (Programma Nazionale Esiti, PNE) in 2010 [7].

The PNE, which started simultaneously for all Italian hospitals, is one of the most complete national healthcare evaluation models, with more than 60 indicators that have been included in other evaluation systems and are considered reliable, valid and clinically relevant [8]. Overall, the system includes a total of 170 outcome or process indicators and provides specific rankings of all hospitals or local health units [9,10]. The main aim, however, is not the ranking itself, it is to identify the providers with the lowest performance and encourage them to conduct quality-of-care audits, and then implement specific strategies to improve their quality [9]. Although some narrative comparisons among national quality systems are available [8], no study has quantitatively evaluated whether the PNE has been able to fulfill its main aim in the hospital setting, improving the performance of the hospitals with the lowest scores. For a subset of outcome indicators, we evaluated whether the performance of the hospitals with the lowest scores in 2016 significantly improved after a realistic amount of time (five years). This would provide an estimate of the role of the PNE indicators as a stimulus for the implementation of effective performance-oriented policies. As a secondary analysis, we investigated the potential association between quality improvement and hospital volume or location.

## 2. Materials and Methods

### 2.1. PNE Characteristics and Methodology

The PNE aims to assess the efficiency, appropriateness and safety of healthcare services provided by the National Health System, as well as equity of access to such services, by calculating and publishing annually a set of indicators of the quality of care [11,12]. In brief, the program calculates and publishes a set of indicators of the quality of care annually. Currently, 170 process and outcome indicators have been outlined concerning the hospital setting, as well as 25 concerning outpatient care, in relation to nine clinical areas: cardio- and cerebrovascular, digestive, musculoskeletal, pediatric, maternity and perinatal, respiratory, oncological, urogenital and infectious diseases [12]. The data were obtained from the official National Healthcare System hospital discharge abstracts (Italian SDO) dataset, and the Tax Register Information System (living status) [10]. The hospital discharge abstracts contained patient demographic data (gender, age), admission and discharge dates, principal diagnosis at discharge and up to 5 secondary diagnoses, medical procedures or surgical interventions (up to 6), access and discharge from emergency department and status at discharge. The National Tax Register was used to collect information on the life status of all patients. Records from different data sources were connected using a deterministic record linkage [10].

Most of the indicators are expressed as ratios (number of patients with a given outcome vs. group of patients at risk), while some other indicators are expressed as survival/waiting time (e.g., time to intervention for surgery after fibula fracture). The analyses were performed both by hospital and by area of residence (local health unit or province of residence). All outcome indicators were adjusted for multiple potential confounders, such as age, sex, disease severity and comorbidities, in order to ensure comparability between hospitals with a different case mix [10].

Among the outcome indicators, 23 computed the mean death rate 30 days after the admission of all patients that were cared for by the same hospital during the calendar year, and computed their (adjusted) relative risk (RR) of death compared to the mean national rate [13]. As an example, for Hospital X, the indicator “Acute Myocardial Infarction (AMI): 30-day mortality” is the proportion of patients—among which all were discharged with a diagnosis of AMI by Hospital X—who died within 30 days from admission. This proportion of patients was then compared to the mean national value, and the relative risk of death of Hospital X patients was computed using multivariable analyses adjusted for the above-mentioned potential confounders. If we assume that the 30-day mortality of Hospital X AMI patients was 12% during the year 2021, and the national mean death rate was 8%, if the Hospital X case mix was identical to the national mean, the (adjusted) RR of death of Hospital X AMI patients would be 1.50.

In addition to the adjusted RR, 95% confidence intervals (CIs) and *p*-values were computed for each hospital, using the same multivariable model and considering the hospital sample size (the number of AMI patients treated during the year). This is essential to reduce the risk of spurious findings, as the *p*-value of the hospitals that treated fewer patients will most likely be higher than 0.05, indicating a low power the comparison between its performance and the national mean value, thus a high degree of uncertainty.

At the end of the process, the hospitals were assigned to one of the following four categories:

- RR > 1.00 and *p* < 0.05 (poor performance—the patients treated by this hospital had a higher likelihood of death than the average Italian patient, and the results are significant, indicating a low level of uncertainty);
- RR > 1.00 and *p* ≥ 0.05 (poor performance, uncertain—the patients treated by this hospital had a higher likelihood of death than the average Italian patient, but the results are not significant);
- RR ≤ 1.00 and *p* ≥ 0.05 (high performance, uncertain—the patients treated by this hospital had an equal or lower likelihood of death than the average Italian patient, but the results are not significant);
- RR ≤ 1.00 and *p* < 0.05 (high performance—the patients treated by this hospital had an equal or lower likelihood of death than the average Italian patient, and the results are significant).

As mentioned above, rather than creating a hospital ranking, the aim of the system is to identify hospitals in the first category, especially those that showed high, significant RRs. Once the hospitals with the lowest performance have been recognized, specific strategies should be implemented to improve their quality of care.

### 2.2. Study Methods

To reduce the risk of information bias, we selected only outcome indicators that measured the risk of death 30 days after a hospital admission for the following common and severe conditions that had the highest burden on the Italian healthcare system [14]:

1. Acute myocardial infarction (AMI);
2. Congestive heart failure (CHF);
3. Stroke;
4. Chronic obstructive pulmonary disease (COPD);
5. Chronic kidney disease (CKD);
6. Femur fracture;
7. Lung cancer;
8. Colon cancer.

For each indicator, we only selected the hospitals with the worst scores in the year 2016 (adjusted RR ≥ 1.30; *p* < 0.05; defined as “Very low performance”), and then traced their performance in the year 2021. We chose a 5-year time span between the first and second measurement, since the PNE results are usually published late in the year (e.g., in September), they are based on the data of the previous calendar year and because the cycles of quality improvement of a complex organization such a hospital rarely last less than two years. Therefore, a long time inevitably passes before the results of the interventions made to improve quality can be seen in a new PNE report.

Based upon its 2021 performance, every hospital was then assigned to one of the following five major groups:

(a) The adjusted RR remained ≥ 1.30, with a *p* < 0.05;
(b) The adjusted RR remained ≥ 1.30, but the *p*-value became equal or higher than 0.05;
(c) The adjusted RR decreased below 1.30, but remained higher than 1.00, regardless of the *p*-value;
(d) The adjusted RR decreased below (or was equal to) 1.00, but the *p*-value was ≥ 0.05;
(e) The adjusted RR decreased below (or was equal to) 1.00, with a *p* < 0.05.

In order to reduce the complexity of the interpretation of multivariate analyses results, the hospitals in the first two groups (a and b) were further classified as “not improved”, while those in the following groups (c, d and e) were classified as “improved”. Importantly, the definition of “improved hospital” only refers to the selected indicators and should not be interpreted as an overall quality improvement of all hospital services. Finally, improvements in RRs should not be conflated with improvements in absolute mortality rates, since these two metrics can move independently.

### 2.3. Data Analysis

The overall proportion of hospitals with improved indicators was reported for each indicator separately, and stratified by geographical area (Northern, Central and Southern Italy) or number of hospital admissions for the selected disease in 2016 (<150, 150–250, >250). Logistic regression was used to evaluate the potential association between hospital improvement and geographical area, volume of admissions and baseline performance (the adjusted RR of death of the hospital in 2016). We performed two sensitivity analyses. First, in order to assess the potential impact of the coding choice of the hospitals, we repeated the multivariate analyses excluding the hospitals of group c, as their performance was still suboptimal and quality improvement was not certain. Second, to evaluate whether the results were dependent on the choice of year, we repeated all analyses using the data of the year 2019. The goodness of fit was checked using the Hosmer–Lemeshow test and the predictive power was assessed using C statistics (area under the receiving operator curve). Statistical significance was defined as a two-sided *p*-value < 0.05, and all analyses were performed using Stata, version 15.0 (Stata Corp., College Station, TX, USA, 2022).

## 3. Results

### 3.1. Overall Performance Variation

As reported in Table 1, during the year 2016, the PNE identified 288 hospitals with a very low performance (adjusted RR ≥ 1.30; *p* < 0.05) in at least one of the selected indicators. Overall, 51.0% (n = 147) of these hospitals showed some degree of improvement during the year 2021, and 27.4% of the hospitals improved so much that the death risk of their patients fell below the national mean value (although this difference was significant for seven hospitals only). Among the 49.0% (n = 141) of hospitals that did not show any improvement, 34.7% (n = 100) showed a significant RR > 1.30 in 2021, thus their patients still carried a mean risk of death that was more than 30% higher than the average Italian patient with the same disease.

### 3.2. Performance by Disease, Geographical Area and Hospital Volumes

**Figure 1:**
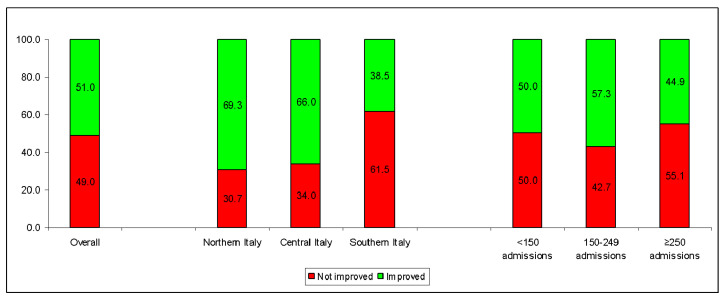
Variation in hospital performance from 2016 to 2021 according to the Italian National Healthcare Outcomes Program (PNE) for selected outcomes, overall and by geographical area or number of hospital admissions (for the selected outcomes) in the year 2016. The sample consists of the 288 hospitals with the poorest performance in the year 2016 for the selected indicators.

While most of the hospitals were able to improve their RR of 30-day mortality of patients hospitalized with colon cancer (81.2%), AMI (67.9%) and femur fracture (66.7%) (Table 1), less than half of the providers improved the RRs of patients hospitalized for lung cancer (83.3%), CKD (57.4%), CHF (55.6%) and COPD (55.6%). The overall proportion of improved indicators also varied largely according to the geographical area and, to a lesser extent, the number of admissions (Table 2 and Figure 1).

While 69.3% and 66.0% of the providers located in Northern and Central Italy, respectively, showed a substantial advancement from 2016 to 2021, only 38.5% of the hospitals in Southern Italy (and the main islands) improved their performances. The three largest regions of the South (Campania, Puglia and Sicily) showed rates of improved indicators lower than 38% (Table 3).

In regard to volumes, hospitals with an intermediate number of admissions (150–249) showed the highest proportion of improved indicators (57.2%; Table 2), while only 45% of the largest providers were able to considerably reduce the risk to their patients.

### 3.3. Multivariate and Sensitivity Analyses

Multivariate analyses, adjusting for the baseline performance in 2016, confirmed univariate results (Table 4). Compared to the hospitals of the Southern regions, those located in Northern and Central Italy had significantly higher odds of improving (OR > 3 for both; *p* < 0.001). Finally, the likelihood of enhancing the performance significantly decreased when the hospital size increased (OR: 0.49; *p* = 0.008 per 20-unit increase in the number of hospitalizations).

In the first sensitivity analysis, when the 68 hospitals with an intermediate performance (adjusted RR of death that decreased below 1.30 but remained ≥1) were excluded from the multivariate model (Table S1), the results did not change substantially, although the association between hospital volume and quality improvement was no longer significant (*p* = 0.065), probably due to a loss in statistical power.

In the second sensitivity analysis, earlier data (year 2019) were used instead of those of the year 2021, and all analyses were repeated (Tables S2–S4). Although the proportion of hospitals with improved indicators was lower (36.1%), especially in Southern regions (24.7%), the results of the multivariate analyses were similar to those obtained when 2021 data were used.

## 4. Discussion

This pre-post analysis evaluated the potential role of the Italian Healthcare Outcomes Program (PNE) through the assessment of the improvement of the hospitals that received the lowest scores in a selection of outcome indicators. The main findings are as follows: (a) more than three years after the communication of the (low) scores, half of the hospitals were able to substantially improve their performance; (b) the rate of improved indicators varied widely by geographical area, with a particularly critical situation in the largest Southern regions, where the majority of the hospitals were unable to recover; (c) the providers with the largest volume showed a lower likelihood of a substantial improvement.

Although every quality assessment system may exert a global positive effect on the delivery of healthcare services through a raise in operator awareness [15], the PNE was developed with the main aim of identifying the most critical providers and guiding them through a quality improvement process. This process requires standardized audits aimed at identifying the causes of low performance [16], and the implementation of several strategies to address these causes, including the development/updating of care pathways based on current guidelines [17], monitoring the adherence to these pathways [18], revising the accreditation indicators to evaluate whether the infrastructure and/or personnel are adequate for the volume of patients and, finally, in the most critical cases, an overall spending review [19]. The final outcome of the process is the enhancement of the underperforming indicators up to the satisfactory threshold [20]. Importantly, while the audits are mandatory, the other quality interventions are only suggested, as the PNE cannot mandate actions at any specific time [11,12].

Overall, for the set of selected indicators, despite the general validity of the PNE program [12], this aim has been achieved only partially, as almost half of the hospitals with the lowest scores did not improve significantly. Although several studies have been published on the reliability and financial impact of hospital quality indicators [21,22,23,24,25], only a few assessments, of which none in Italy, are available on the performance of national quality assessment systems, and comparing the results is challenging due to the diversity of healthcare systems and methodologies. Overall, our findings are consistent with the available body of literature, where studies on the impact of quality programs on healthcare providers often yield similarly mixed results [26,27,28]. In a time-trend analysis from 2006 to 2014, Pross et al. assessed the evolution of German hospitals, categorizing them into quintiles based on several outcomes [26]. This study identified positive trends in the quality of hip replacement surgery, along with a reduction in the 30-day mortality rates for stroke and AMI. However, it also showed negative trends in 90-day readmission rates for stroke and AMI, and inpatient mortality for percutaneous coronary interventions. According to the authors, these findings highlight that mere reporting and measurement of hospital outcomes, without specific policies and/or interventions, may only lead to partial rather than systematic quality improvements [26]. Another German, more recent, pre-post analysis (2015–2021) evaluated the impact of adopting the IQTIG (Federal Institute for Quality Assurance and Transparency in Healthcare) quality assessment program using 11 indicators in the gynecological, obstetrics and breast surgery areas across half of the national hospitals [27]. Although the analysis showed improvement in the initial two years of the program, this was attributed to a better coding accuracy, as one of IQTIG’s objectives was to consult policy makers to plan hospital capacity, leading to the exclusion of continuously underperforming hospitals from being reimbursed by the statutory health insurance. Some federal states opted not to include these data in their planning, leading to limited changes in hospital planning.

More positive, although less comparable findings were reported in studies evaluating the impact of the quality assessment systems in Taiwan [28] and Iran [29], while no improvements, or very mixed results were reported in the USA by the Centers for Medicare and Medicaid Services (CMS) [30]. Between 2012 and 2014, the CMS started three programs that adjusted Medicare hospital payments through rewards and penalties, aiming to reduce readmissions and hospital-acquired conditions, and to promote value-based purchasing [31]. A pre-post evaluation (2007–2016) of administrative datasets from 14 States found extremely mixed results in the hospital quality and safety indicators that were targeted by the three programs [30]. Interestingly, another assessment found penalties to be associated with environmental and social characteristics that hospitals cannot control—i.e., medical complexity, uncompensated care and the portion of population who live alone [31]. While a direct comparison with our work is impossible, these findings from the USA are consistent with a preliminary analysis of the performance of Italian hospitals, which suggested that prospective reimbursements have the potential to improve efficiency, but they do not necessarily improve quality of care [32]. This is confirmed by a systematic review which found uncertain evidence that pay-for-performance programs have an impact on patient outcomes, quality of care or resource use [33].

As mentioned, the northern and central Italian regions showed a substantially higher rate of improved indicators compared to the southern ones, indicating an unequal ability to recover of the latter regions [34], and confirming the well-documented disparities between these areas, often referred to as the “North-South Gap” [35], that has been documented through public health and health service accessibility indicators [36]. This healthcare inter-regional inequality has been related to a series of substantial, historical socio-economic differences between the northern and southern regions, as well as the current federalist framework of the Italian NHS, which awards large degrees of freedom to the individual regional governments regarding the organizational models and strategies used [37].

Possibly due to a higher level of specialization and compliance with evidence-based processes, larger hospitals repeatedly showed higher quality scores [38], even in a dedicated analysis based on PNE indicators [39]. Notably, compared to the hospitals with the lowest volume, the largest hospitals showed a significantly lower likelihood of improvement in the selected indicators, which may easily reflect the greater challenges in managing organizational changes in bigger facilities, with a diversified case-mix and complex bureaucracy [39].

The strengths of the study are the official, validated data on all Italian hospitals, and the stability of the national mean value of each of the selected indicators from 2016 to 2021, which prevents a confounding effect due to a background trend of improvement/worsening of the selected outcomes. The study also has limitations that must be considered when interpreting the results. First, although the selected indicators were chosen to minimize the risk of information bias, the coding accuracy can inevitably impact the scores [21]. Second, although the indicators represent eight epidemiologically relevant diseases, they do not encompass all aspects of hospital performance. Third, since statistical significance is influenced by the sample size, very small hospitals could not be included in the baseline list even if they obtained very low scores in 2016. Fourth, the period of study was characterized by the concomitant COVID-19 pandemic, which inevitably had an impact on hospitals’ capacity to improve. Finally, this study aims to provide an estimate of the role of PNE indicators as a stimulus for the implementation of performance-oriented policies. Although hospitals with suboptimal performances are actively encouraged to implement improvement policies, the study design cannot provide direct evidence that the improvements in 2021 were caused by interventions stimulated by PNE results.

## 5. Conclusions

This study shows that half of the hospitals with the poorest performance based on selected outcome indicators of the Italian National Healthcare Outcomes Program (PNE) improved from 2016 to 2021. A large North–South disparity was observed, with the majority of the providers located in the largest Southern regions, that were unable to substantially improve. Further studies are necessary to evaluate the causal relationship between the PNE ranking and quality outcome indicators.

## Figures and Tables

**Table 1:** Overall variation of performance (years 2016–2021) in selected indicators of the 288 hospitals with the poorest performance * in the year 2016 according to the Italian National Healthcare Outcomes Program (PNE).

	All Outcomes	AMI	CHF	Stroke	COPD	CKD	Femur Fracture	Colon Cancer	Lung Cancer
	n = 288	n = 28	n = 72	n = 32	n = 36	n = 68	n = 30	n = 16	n = 6
	%	%	%	%	%	%	%	%	%
Overall performance									
Improved	51.0	67.9	44.4	53.1	44.4	42.6	66.7	81.2	16.7
− Adj. RR decreased below 1.30 but remained > 1	23.6	32.1	25.0	21.9	13.9	20.6	30.0	37.5	0.0
− Adj. RR decreased ≤ 1.00, *p* ≥ 0.05	25.0	28.6	16.7	28.1	30.6	19.1	36.7	43.8	16.7
− Adj. RR decreased < 1.00, *p* < 0.05	2.4	7.1	2.8	3.1	0.0	2.9	0.0	0.0	0.0
Did not improve	49.0	32.1	55.6	46.9	55.6	57.4	33.3	18.8	83.3
− Adj. RR remained ≥ 1.30, *p* < 0.05	34.7	17.9	34.7	25.0	44.4	52.9	23.3	12.5	16.7
− Adj. RR remained ≥ 1.30, *p* ≥ 0.05	14.2	14.3	20.8	21.9	11.1	4.4	10.0	6.3	66.7

* Hospitals that, in 2016, showed a statistically significant adjusted RR of mortality in the 30 days after admission for at least one of the selected outcomes higher than 1.30 (compared to the national mean value). Adj. = Adjusted. AMI = 30-day mortality rate from the day of hospital admission for acute myocardial infarction. CHF = 30-day mortality rate from the day of hospital admission for congestive heart failure. Stroke = 30-day mortality rate from the day of hospital admission for stroke. COPD = 30-day mortality rate from the day of hospital admission for chronic obstructive pulmonary disease. CKD = 30-day mortality rate from the day of hospital admission for chronic kidney disease. Femur F. = 30-day mortality rate from the day of hospital admission for a fracture of the femur. Colon C. = 30-day mortality rate from the day of hospital admission for colon cancer. Lung C. = 30-day mortality rate from the day of hospital admission for lung cancer.

**Table 2:** Variation in performance from 2016 to 2021 for selected indicators of the 288 hospitals with the poorest performance * in the year 2016, according to the Italian National Healthcare Outcomes Program (PNE), by selected variables.

	aRR ≥ 1.30, *p* < 0.05	aRR ≥ 1.30, *p* ≥ 0.05	1 < aRR < 1.30	aRR ≤ 1.00, *p* ≥ 0.05	aRR < 1.00, *p* < 0.05
	% (n)	% (n)	% (n)	% (n)	% (n)
**Geographical area**					
− Northern Italy	17.3 (13)	13.3 (10)	28.0 (21)	40.0 (30)	1.3 (1)
− Central Italy	27.7 (13)	6.4 (3)	19.2 (9)	36.2 (17)	10.6 (5)
− Southern Italy	44.6 (74)	16.9 (28)	22.9 (38)	15.1 (25)	0.6 (1)
N. of hospital admissions ** in 2016					
<150	35.4 (34)	14.6 (14)	18.8 (18)	29.2 (28)	2.1 (2)
150–249	26.2 (27)	16.5 (17)	25.2 (26)	29.1 (30)	2.9 (3)
≥250	43.8 (39)	11.2 (10)	27.0 (24)	15.7 (14)	2.3 (2)

* Hospitals that, in the year 2016, showed a statistically significant adjusted RR of mortality in the 30 days after admission for at least one of the selected outcomes higher than 1.30 (compared to the national mean value). ** Number of admissions for the selected outcomes.

**Table 3:** Variation in the performance (years 2016–2021) in selected indicators of the 288 hospitals with the poorest performance * in the year 2016, according to the Italian National Healthcare Outcomes Program (PNE), by region.

Regions	Not Improved	Total
	% (n)	n
Piemonte	21.1	19
Liguria	0.0	8
Lombardia	36.8	19
Veneto	50.0	14
Friuli Venezia Giulia	50.0	4
Emilia Romagna	27.3	11
Toscana	21.4	14
Umbria	-	-
Marche	20.0	5
Lazio	42.9	28
Abruzzo	58.3	12
Molise	50.0	6
Basilicata	40.0	5
Campania	68.0	50
Calabria	58.8	17
Puglia	62.2	39
Sicilia	68.8	32
Sardegna	14.3	7
Total (Italy)	49.0	288

* Hospitals that, in 2016, showed a statistically significant adjusted RR of mortality in the 30 days after admission for at least one of the selected outcomes higher than 1.30 (compared to the national mean value).

**Table 4:** Logistic regression model predicting a substantial improvement in the selected indicators (any outcome) from 2016 to 2021, according to selected characteristics.

	OR	(95% CI)	*p*
Geographical area			
− Southern Italy	1 (Ref. cat.)	--	--
− Northern Italy	3.37	(1.86–6.11)	<0.001
− Central Italy	3.28	(1.63–6.61)	0.001
N. of hospital admissions in the year 2016, 20-admission increase	0.49	(0.29–0.83)	0.008
Adjusted RR in the year 2016, 1-unit increase	0.94	(0.91–0.98)	0.007

Ref. cat. = reference category. OR = odds ratio. CI = confidence interval.

## Data Availability

All the data is publicly available on the official PNE website: https://pne.agenas.it/home (accessed on 5 February 2024).

## Supplementary Materials

The following supporting information can be downloaded at: https://www.mdpi.com/article/10.3390/healthcare12040431/s1, Table S1: Logistic regression model predicting a substantial performance improvement in the selected indicators (any outcome) from 2016 to 2021, according to selected characteristics, and excluding the 68 hospitals with a 2021 adjusted relative risk that decreased below 1.30, but remained >1.00. Table S2: Overall variation in the performance (years 2016–2019) of selected indicators of the 288 hospitals with the poorest performance * in the year 2016, according to the Italian National Healthcare Outcomes Program (PNE). Table S3: Variation in the performance from 2016 to 2019 for selected indicators of the 288 hospitals with the poorest performance * in the year 2016, according to the Italian National Healthcare Outcomes Program (PNE), by selected variables. Table S4: Logistic regression model predicting a substantial performance improvement in the selected indicators (any outcome) from 2016 to 2019, according to selected characteristics.
