# Supplementary material for: Impact of the Italian Healthcare Outcomes Program (PNE) on the Care Quality of the Poorest Performing Hospitals
Source: Healthcare (Basel). 2024 Feb 7;12(4):431. doi: 10.3390/healthcare12040431 (PMC10887701; doi:10.3390/healthcare12040431)
Supplement: Supplementary file 1 [file healthcare-12-00431-s001.zip › healthcare-2850157-supplementary.pdf]

Table S1. Logistic regression model predicting a substantial performance improvement in the selected indicators (any outcome) from 2016 to 2021, according to selected characteristics, and excluding the 68 hospitals with a 2021 Adjusted Relative Risk that decreased below 1.30, but remained >1.00.

	**OR**	**(95% CI)**	**p**
**Geographical area**			
- Southern Italy	1 (Ref. cat.)	--	--
- Northern Italy	4.89	(2.39-9.98)	<0.001
- Central Italy	6.82	(2.97-15.65)	<0.001
N. of hospital admissions in the year 2016, 20-admission increase	0.56	(0.30-1.04)	0.065
Adjusted RR in the year 2016, 1-unit increase	0.91	(0.86-0.97)	0.003

Ref. cat. = Reference category. OR = Odds Ratio. CI = Confidence Interval.

Table S2. Overall variation of the performance (years 2016-2019) in selected indicators of the 288 hospitals with the poorest performance* in the year 2016, according to the Italian National Healthcare Outcomes Programme (PNE).

	**All outcomes**	**AMI**	**CHF**	**Stroke**	**COPD**	**CKD**	**Femur Fracture**	**Colon Cancer**	**Lung Cancer**
	n=288	n=28	n=72	n=32	n=36	n=68	n=30	n=16	n=6
	%	%	%	%	%	%	%	%	%
Overall performance									
Improved	36.1	53.6	36.1	28.1	33.3	29.4	63.3	18.8	0.0
- Adj. RR decreased below 1.30 but remained >1	21.5	32.1	22.2	18.7	16.7	16.2	43.3	6.3	0.0
- Adj. RR decreased ≤1.00, p≥0.05	13.6	17.9	13.9	9.4	16.7	10.3	20.0	12.5	0.0
- Adj. RR decreased <1.00, p<0.05	1.0	3.6	0.0	0.0	0.0	2.9	0.0	0.0	0.0
Did not improve	63.9	46.4	63.9	71.9	66.7	70.6	36.7	81.3	100.0
- Adj. RR remained ≥1.30, p<0.05	31.3	25.0	30.6	40.6	22.2	26.5	20.0	68.8	83.3
- Adj. RR remained ≥1.30, p≥0.05	32.6	21.4	33.3	31.3	44.4	44.1	16.7	12.5	16.7

* Hospitals that, in 2016, showed a statistically significant adjusted RR of mortality in the 30 days after admission for at least one of the selected outcomes higher than 1.30 (as compared to the national mean value).
Adj. = Adjusted. AMI = 30-days mortality from the day of the hospital admission for acute myocardial infarction. CHF = 30-days mortality from the day of the hospital admission for congestive heart failure. Stroke = 30-days mortality from the day of the hospital admission for stroke. COPD = 30-days mortality from the day of the hospital admission for Chronic Obstructive Pulmonary Disease. CKD = 30-days mortality from the day of the hospital admission for chronic kidney disease. Femur F. = 30-days mortality from the day of the hospital admission for a fracture of the neck of femur. Colon C. = 30-days mortality from the day of the hospital admission for colon cancer. Lung C. = 30-days mortality from the day of the hospital admission for lung cancer. The data from 14 hospitals were missing in the year 2019: according to an Intention-To-Treat logic, they have been assigned to the category "Not improved, not significant".

Table S3. Variation in the performance from 2016 to 2019 for selected indicators of the 288 hospitals with the poorest performance* in the year 2016, according to the Italian National Healthcare Outcomes Programme (PNE), by selected variables.

	**aRR≥1.30, p<0.05** % (n)	**aRR≥1.30, p≥0.05** % (n)	**1<aRR<1.30** % (n)	**aRR≤1.00, p≥0.05** % (n)	**aRR<1.00, p<0.05** % (n)
**Geographical area**					
- Northern Italy	24.0 (18)	29.3 (22)	34.7 (26)	12.0 (9)	0.0 (0)
- Central Italy	25.5 (12)	14.9 (7)	29.8 (14)	25.5 (12)	4.3 (2)
- Southern Italy	38.6 (64)	36.7 (61)	13.3 (22)	10.8 (18)	0.6 (1)
**N. of hospital admissions** in 2016**					
<150	25.0 (24)	33.3 (32)	25.0 (24)	15.6 (15)	1.0 (1)
150-249	28.2 (29)	36.9 (38)	17.5 (18)	16.5 (17)	1.0 (1)
≥250	46.1 (41)	22.5 (20)	22.5 (20)	7.9 (7)	1.0 (1)

* Hospitals that, in the year 2016, showed a statistically significant adjusted RR of mortality in the 30 days after admission for at least one of the selected outcomes higher than 1.30 (as compared to the national mean value)
** Number of admissions for the selected outcomes.
The data from 14 hospitals were missing in the year 2019: according to an Intention-To-Treat logic, they have been assigned to the category "Not improved, not significant".

Table S4. Logistic regression model predicting a substantial performance improvement in the selected indicators (any outcome) from 2016 to 2019, according to selected characteristics.

	OR	(95% CI)	p
**Geographical area**			
- Southern Italy	1 (Ref. cat.)	--	--
- Northern Italy	2.38	(1.30-4.34)	0.005
- Central Italy	5.24	(2.53-10.87)	<0.001
N. of hospital admissions in the year 2016, 20-admission increase	0.25	(0.12-0.51)	<0.001
Adjusted RR in the year 2016, 1-unit increase	0.92	(0.87-0.96)	0.001

Ref. cat. = Reference category. OR = Odds Ratio. CI = Confidence Interval.
